# Cultural efficacy as a novel component of understanding linkages between culture and mental health in Indigenous communities

**DOI:** 10.1002/ajcp.12594

**Published:** 2022-03-14

**Authors:** Miigis B. Gonzalez, Kelley J. Sittner, Melissa L. Walls

**Affiliations:** ^1^ Johns Hopkins Bloomberg School of Public Health, Center for American Indian Health, Great Lakes Hub Duluth Minnesota USA; ^2^ Department of Sociology Oklahoma State University Stillwater Oklahoma USA

**Keywords:** culture, enculturation, Indigenous peoples, mental health, wellbeing

## Abstract

We used a novel measure of *cultural efficacy* to examine empirical pathways between enculturation, efficacy, and two wellbeing outcomes. Cultural factors are not consistently linked to better wellbeing in the academic literature despite widespread understanding of these processes in Indigenous communities. Healing pathways is a community‐based participatory study with eight reservations/reserves in the upper Midwest and Canada. This study uses data collected in 2017–2018 (*n* = 453, 58.1% women, mean age = 26.3 years) and structural equation modeling to test the relationships between enculturation, *cultural efficacy*, and mental health. The direct effect of enculturation on anxiety was positive. The indirect effect of enculturation via *cultural efficacy* was negatively associated with anxiety and positively associated with positive mental health. *Cultural efficacy* is an important linking variable through which the protective effects of culture manifest. The complex nature of culture must be met with innovative measures and deep understanding of Indigenous peoples to fully capture the protective role of culture.

## Highlights

- Empirical evidence often fails to reflect the innate wisdom within Indigenous communities.
- *Cultural efficacy* considers the colonial impact on one's personal agency to engage culturally.
- *Cultural efficacy* is important to consider when promoting Indigenous mental wellbeing.

## BACKGROUND: THE STATE OF CULTURAL REVITALIZATION

Indigenous culture affects mental health among Indigenous peoples in unique ways, underscoring the importance of novel measures to expand and refine empirical investigations of this relationship. Historical trauma, racism, microaggressions, discrimination, and systemic oppression deeply impact Indigenous communities (Brave Heart & DeBruyn, 1998; Kirmayer et al., 2014), directly affecting mental health (Brockie et al., 2015; Whitbeck, Adams, et al., 2004; Whitbeck et al., 2002) and complicating the relationship between Indigenous cultural factors and wellbeing (Gone, 2014). In some studies, Indigenous culture is shown to be protective against these historical and contemporary traumas (Spence et al., 2016; Wexler, 2014) and mental health problems (Ursula Running et al., 2018; Wolsko et al., 2007). Yet, evidence from other studies suggests a more complicated postcolonial context in which historical trauma, historical losses, and discrimination obscure the protective effects of Indigenous culture (Soto et al., 2015; Walls et al., 2016). The contemporary circumstances of Indigenous communities are complex and deserve deeper interrogation of cultural measures to better support Indigenous cultural revitalization and to enhance the healing powers of Indigenous traditional practices, values, and beliefs.

Cultural revitalization is a strategic priority in Indigenous community efforts to reclaim health and healing for their own peoples (Kagawa‐Singer et al., 2014; Kral et al., 2011; Schultz, Cattaneo, et al., 2016). Colonial oppression and cultural genocide assaulted traditional ways of knowing by way of government‐sanctioned policies including boarding schools, reservation formation, and forced relocation. The devastating effects of such historically traumatic experiences are fundamental determinants of contemporary Indigenous health inequities (King et al., 2009; Wolsko et al., 2007). Current generations of Indigenous peoples are working diligently with their elders to redress these harms through reclamation of Indigenous practices, beliefs, and values (Schultz, Walters, et al., 2016; Ullrich, 2019).

In this vein, culturally based, community‐driven approaches to public health research and practice are necessary steps toward achieving Indigenous health equity (Barker et al., 2017; Kading et al., 2015; Walters et al., 2019). There is a relatively recent body of evidence that Indigenous cultural factors offer health‐promoting benefits (e.g., Kaholokula et al., 2018; Mckinley et al., 2020). From an empirical perspective, however, documenting relationships between culture and wellness are complicated by measurement specificity and validity issues, interactions among culturally salient risk and protective factors, and multigenerational diversity in Indigenous conceptions of “traditional culture” (Walls et al., 2016). In short, science lags behind Indigenous wisdom regarding the health‐promoting effects of culture. Traditional ways of being—eating, parenting, engaging with community, and healing, for example—are naturally protective against heart disease, diabetes, suicide, and other major health issues that disproportionally plague Indigenous communities today (Cho et al., 2014; Herne et al., 2014; Veazie et al., 2014). Innovations in the conceptualization and measurement of Indigenous cultural concepts are critical to advancing scientific understanding of these processes. Each Indigenous community and individual is on its own trajectory of cultural revitalization (Bennett & Liu, 2018; Hall & Fenelon, 2004). The ways in which communities, families, and individuals are focusing cultural revitalization efforts are as broad as definitions of culture itself. In this study, we use the term *enculturation* (e.g., a process or degree of embedment in one's ethnic/cultural group; Zimmerman et al. [1996) to include language use, food practices and harvesting, ceremonial practices and spiritual beliefs, and engagement in cultural arts. As Indigenous community revitalization efforts expand processes of enculturation, future generations may find ease in accessing cultural resources. Today, however, lingering consequences of colonization and historical cultural losses including discomfort related to cultural knowledge and protocols, and unfamiliarity with/lack of access to ceremonial leaders can result in disconnection from cultural sources of strength (Lucero, 2014). In attempt to operationalize this phenomenon, we employ a novel measure of *cultural efficacy*. This measure includes indicators related to confidence in ability to learn and who to ask to learn cultural ways, satisfaction with cultural engagement opportunities, and desire to give back and contribute to culture and community. The measure is also one attempt to consider the current state of cultural revitalization. Its development was driven by comprehensive community input and is supported by theory, as we elaborate upon below.

## **THEORETICAL SUPPORT:** *CULTURAL EFFICACY* **AS AN INDICATOR OF WELLBEING**

Below, we briefly describe three major theories (i.e., Bandura's self‐efficacy theory, empowerment theories, and Campinha‐Bacote's cultural competence model) and review prior studies that informed our process of conceptualization and measurement of *cultural efficacy*. First, Bandura described self‐efficacy as one's belief in their ability to engage in a given activity (Bandura, 1977). Conventional measures of self‐efficacy have been shown to improve health behaviors including physical activity (Olander et al., 2013), medication adherence (Náfrádi et al., 2017) and substance use treatment (Kadden & Litt, 2011). Coupling the growing body of literature that supports culture as healing (Kagawa‐Singer et al., 2014) with teachings of Indigenous Elders, our study considers enculturation as the health behavior or “treatment” necessary for improved health and wellness among Indigenous populations. As a result of colonial destruction and contemporary systemic oppression (Gone, 2013), this behavior is no longer easily acquired. In an attempt to measure this lack of ease we broadened the concept of self‐efficacy to consider *cultural efficacy*, which includes assessment of one's confidence to learn cultural ways, engage in cultural activities, and acquire traditional knowledge.

Second, we felt it important that our operationalization of *cultural efficacy* included an assessment of purpose or motivation towards the health behavior (i.e., enculturation), which is supported by empowerment theories. Empowerment replaces ideas of helplessness to focus on the positive effects of exerting personal control over one's life (Zimmerman, 1990). For example, Shankar (2015, 2018) found that increasing personal agency improved economic conditions and health‐seeking behaviors among participants. Control over one's ability to engage culturally and to be well may be encouraged via concepts described within empowerment theories like knowledge, competence, goal identification, and consideration of the impact of goal attainment on one's community (Cattaneo & Chapman, 2010; Zimmerman, 1990). Linking empowerment concepts to the goals of Indigenous young adults to contribute to Indigenous society (Kading et al., 2019), our conceptualization of *cultural efficacy* includes items pertaining to contribution and role modeling.

Third, we draw upon five components of Campinha‐Bacote's cultural competence model for effective delivery of healthcare services within communities of color (Campinha‐Bacote, 2002) to conceptually frame this study. Although the components are not designed for individuals working/living within their own culture, the model embraces the importance of seeking and obtaining cultural knowledge, knowing how to appropriately navigate cultural spaces, engaging culturally, and having a genuine desire to learn more. Seeking cultural “services” within one's own culture undoubtedly requires similar, if not deeper, sets of knowledge and skills.

The term *cultural efficacy* has been coined in one prior study that grounds and supports the ideas within our work. Houkamau and Sibley (2011) defined *cultural efficacy* as perceptions of personal resources to effectively and appropriately engage with Indigenous (in their case, Māori) cultural and social contexts. They found a positive association between cultural efficacy and personal life satisfaction indicators (i.e., standard of living, health, achievements, relationships, community connection, and future security) among Māori participants. Their definition of cultural efficacy validates our own understanding of efficacy within a cultural framework of health. Additionally, their results support our investigation of potential indirect pathways between culture and wellbeing via cultural efficacy. In other words, our project assumes that *cultural efficacy* not only affects mental health but also offers another explanation for why Indigenous culture has been shown in some studies to be negatively associated with indicators of health (Walls et al., 2016). Our project thus expands the work of Houkamau and Sibley (2011) to examine the relevance of *cultural efficacy* for other Indigenous peoples.

Our team also found support for the importance of assessing cultural efficacy in formative work leading to the current study. For instance, we generated an Indigenous (Anishinaabe) framework of health through group concept mapping processes and intensive community feedback. The framework defined how Anishinaabe young adults perceive wellbeing (Kading et al., 2019). Anishinaabe culture permeated the resulting themes, including honoring ancestors and the earth, purposeful engagement in Anishinaabe culture, acquiring and sharing traditional knowledge, and having pride in being Anishinaabe even amongst pressures of assimilation, discrimination, and microaggressions. Additionally, results highlighted taking care of oneself, being able to set and accomplish goals, looking into the future, and having obligation to community. Major outcomes of that work emphasized the importance of culture for Indigenous wellbeing and prioritized the individual's ability and drive to “be well,” thus underscoring the concept of personal agency to purposefully engage in wellbeing behaviors, that is, cultural activities (Kading et al., 2019).

**Figure 1:**
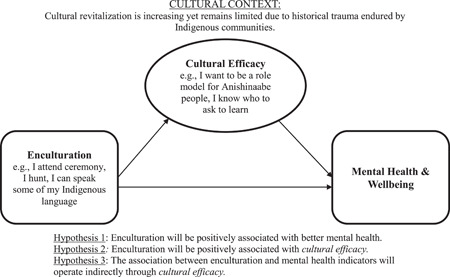
Conceptual model of *cultural efficacy*

In conclusion, *cultural efficacy* may be a compelling contributor to Indigenous health given the complexities of postcolonial Indigenous experiences of culture (Walls et al., 2016). That is, personal perceptions and confidence engaging with Indigenous culture may be an important pathway through which enculturation confers health‐promoting effects. Figure 1 provides a conceptual model for the proposed analyses informed by the theoretical and empirical foundations described above. As shown, we expect in a sample of Indigenous young adults:

Hypothesis 1 Enculturation will be positively associated with better mental health.

Hypothesis 2 Enculturation will be positively associated with *Cultural Efficacy*.

Hypothesis 3 The association between enculturation and mental health indicators will operate indirectly through *cultural efficacy*.

## DATA AND METHODS

### Participants

The sample consisted of 453 young adults, 58.1% of whom were women, with a mean age of 26.3 years. Approximately 67% of the sample lived on reservation/reserve lands and 31.3% had at least some postsecondary education. Approximately 43.5% of the young adults were employed full‐time, 15.2% were employed part‐time, and 35.3% were unemployed. The median personal income was $12,500. Almost half of the sample (47.2%) were married or cohabiting, and 85.4% identified as heterosexual. Of those who shared another sexual orientation, the largest group specified bisexual (8.6%).

### Procedures

These data come from the Healing Pathways (HPs) Project, a community‐based participatory study in the upper Midwest and Canada, conducted through partnerships between eight reservation/reserve communities and a university‐based research team. Before the start of the study, a list of families of age‐eligible, tribally enrolled children living within 50 miles of each reservation/reserve was obtained. Project interviewers attempted to contact all of those families and recruit them to the study; the baseline response rate was 79.4%, or 735 adolescents. The original longitudinal panel study began in 2002 and consisted of eight annual interviews with the adolescents and at least one caregiver (Waves 1–8). The second part of the study was a 3‐year follow‐up (Waves 9–11) with the original adolescent participants, now adults (Wave 9 *n* = 453, mean age = 26.3 years). The current analysis used Wave 9 data, the first wave in which all of the focal variables were measured, collected between 2017 and 2018.

Community Research Councils (CRCs) from each location were established to collaboratively develop study procedures including survey development, measurement selection, questionnaire review, pilot testing surveys, recruiting, interviewing, and collaboratively (with university team) hiring project interviewers, setting hourly pay rates for project staff, and identifying priorities for data dissemination. All interviewers were members of the communities within which they worked. The university team developed and hosted in‐depth, multiday trainings focused on survey interview techniques, confidentiality, and research ethics in Indigenous communities. These trainings included dyadic materials, role‐plays, round‐robin survey practice sessions, and group discussions/cross‐training of university team members to consider methods in the context of interviewers' lived experience and wisdom within their own communities. After in‐person trainings, interviewers were assigned “practice interviews,” then completed a phone‐based certification process with university staff before engaging with project participants. Interviewers worked to complete in‐person, interviewer‐administered computer‐assisted and paper‐and‐pencil survey interviews at each wave of the study. Additional study design details can be found in (Walls et al., 2020; Whitbeck et al., 2014). All procedures were approved by approved by Johns Hopkins University IRB.

### Measures

For univariate and bivariate analyses, we constructed composite scales for the focal variables, either by summarizing (i.e., enculturation, flourishing, anxiety) or averaging (cultural efficacy) the items in each scale. Table 1 includes the descriptive statistics and Cronbach's *α*. All latent variable factor loadings and descriptive statistics are shown in Appendix A.

**Table 1:** Descriptive statistics and zero‐order correlations of study variables

	1	2	3	4	5	6	7	8
1. Flourishing	1.00							
2. Anxiety	−.31***	1.00						
3. Cultural efficacy	.28***	−.13**	1.00					
4. Enculturation	.14**	.10*	.42***	1.00				
5. Sex	.00	.22***	‐.01	.08	1.00			
6. On versus off	−.11*	.02	.14**	.01	−.10*	1.00		
7. Remote	−.04	−.09	.14**	.08	−.01	−.04	1.00	
8. Education	.19***	.04	.04	.16**	.12*	−.27***	‐.13**	1.00
Mean	24.00	5.31	2.74	0.00	.58	.67	.12	.31
SD	4.55	5.53	0.62	3.68				
Range	4.00–32.00	0.00–21.00	1.00–4.00	−7.40–11.51				
Cronbach's *α*	.89	.93	.70	.79				

*Note*: Enculturation is an index of standardized traditional and spiritual activities checklists, frequency of traditional activities participation, importance of spirituality, and language use. Sex coded 1 = female, 0 = male; on versus off coded 1 = on reservation/reserve, 0 = off reservation/reserve; remote location coded 1 = remote, 0 = not remote; education coded 1 = at least some postsecondary education, 0 = no postsecondary education.

*** *p* < .001

** *p* < .01

* *p* < .05.

Enculturation was a multidimensional latent construct consisting of different aspects of traditional culture (Whitbeck, Chen, et al., 2004; Whitbeck et al., 2002). Traditional activity involvement was a checklist of 17 activities engaged in during the past year (e.g., beading, hunting; 0 = *no* and 1 = *yes*); affirmative responses were summed together. Language fluency consisted of five questions on the traditional language (e.g., can you understand some of the Anishinaabe language, can you speak Anishinaabe conversationally; coded 0 = *no*, 1 = *yes*). Responses were summed to assess language comprehension and fluency. Three aspects of spirituality were included. First, spiritual activities, a checklist of 13 activities in the past year (e.g., offered tobacco, attended ceremonial feasts; 0 = *no* and 1 = *yes*), was used to create a summary measure of spiritual activities by adding the affirmative responses in the checklist. Second, the frequency of participating was assessed with one item on a 6‐point scale, ranging from 0(*never*) to and 6 (*every day*). Third, we assessed the importance of spiritual values in leading one's life with a single item, with responses ranging from 0 (*not at all important*) to 3 (*very important*). Each component of enculturation was standardized before summing into the overall scale.

Before Wave 9 surveys, the entire research team (community‐ and university‐based members) met for a two day in‐person meeting to discuss measurement priorities for upcoming assessments. Much of our discussion centered around conceptualizing “culture” from an Indigenous perspective. Among the themes of the discussion was attention to angst surrounding cultural loss and desires to engage and reclaim cultural ways. Based on this, five members of the university team (two native, three nonnative) searched existing literature and measures that assessed these ideas. Ultimately, our operationalization of *cultural efficacy* was developed using five items adapted from the Native Wellness Assessment Self‐Report Form (Thunderbird Partnership Foundation, 2015). The adaptation process involved: (a) university team members reviewing/pulling items from the assessment relevant to the concept of *cultural efficacy*; (b) community team members reviewing, removing, or revising items; and (c) piloting the measure with a convenience sample of Indigenous young adults who gave input on their comprehension and perceived meaning of measurement items. For actual assessments, HPs participants were asked whether they agreed or disagreed with five statements: (1) I feel confident about my ability to learn Anishinaabe cultural ways; (2) many of the things I do are to give back to Anishinaabe people; (3) I am satisfied with my opportunities to engage in Anishinaabe cultural ways; (4) I feel sure about who to ask to learn about Anishinaabe cultural ways; (5) I want to be a role model for other Anishinaabe people. Response options ranged from 0 (*strongly disagree*) to 4 (*strongly agree*).

We included two mental health outcomes that measured positive and negative mental health. *Anxiety* symptoms were measured with the Generalized Anxiety Disorder Scale‐7 (GAD‐7; Spitzer et al., 2006), a seven‐item screening instrument to assess the frequency of anxiety symptomatology in the past 2 weeks. Response options ranged from 0 (*not at all*)to 3 (*nearly every day*). The GAD‐7 has been found to be a valid measure of anxiety symptom severity for different age, race/ethnicity, and gender groups (Parkerson et al., 2015; Sriken et al., 2021; Wild et al., 2014), and has been used with Indigenous samples (Chahar Mahali et al., 2020; McKinley et al., 2021). *Flourishing* (Diener et al., 2010) was measured with eight statements evaluating self‐perceived success in important domains with a 5‐item Likert scale 0 (*strongly disagree*)to 4 (*strongly agree*). The flourishing scale has been validated across age groups (Howell & Buro, 2015; Romano et al., 2020) and has been applied in international Indigenous contexts (Hone et al., 2014; Ritchie et al., 2014).

We controlled for basic demographic characteristics in the final analysis. *Sex* was coded as 1 = female, 0 = male. *Education* was a categorical variable in which participants self‐reported their highest level of education; this was dichotomized for analysis to 0 = *no postsecondary education*; 1 = *at least some postsecondary education*. We accounted for location differences with indicators of proximity to reservation/reserve lands (0 = *living off reservation/reserve*; 1 = *living on reservation/reserve*) and relative geographic isolation from other sites (0 = *not remote*; 1 = *remote*).

### Analytic strategy

For descriptive statistics and zero‐order correlations, we used SPSS version 26 to construct scales for the focal variables. Structural equation modeling in Mplus 7.1 (Muthén & Muthén, 1998) with maximum likelihood estimation and robust standard errors was used to test our hypotheses about enculturation, *cultural efficacy*, and mental health. We began by using confirmatory factor analysis to construct each latent variable separately. Model fit was judged using individual values and converging evidence across comparative fit index (CFI), Tucker–Lewis index (TLI), and root mean square error of approximation (RMSEA), with CFI and TLI close to or greater than 0.95, and RMSEA close to or less than 0.06 (Hu & Bentler, 1999). Modification indices were examined to identify missing correlations that would improve model fit (modification indices > 30). For our measure of cultural efficacy, we first examined a one‐factor model, which had poor fit to the data (*χ* ^2^ = 32.86, *df* = 5, *p* < .000; CFI = 0.88; TLI = 0.76; RMSEA = 0.11). We then examined two‐factor models (Items 2 and 5 as the first factor; Items 1, 3, and 4 as the second). The model fit was significantly better (*χ* ^2^ = 4.56, *df* = 3, *p* = .21; CFI = 0.99; TLI = 0.98; RMSEA = .03), and was used in scanning electron microscopy analyses. Our young adult enculturation latent construct also had good fit to the data (*χ* ^2^ = 11.94, *df* = 4, *p* = .02; CFI = 0.99; TLI = 0.97; RMSEA = 0.07). Psychometric studies of anxiety and flourishing each support a single factor structure, both of which provided acceptable model fit in the current study. After fitting each latent variable individually, we constructed a measurement model with correlations among all latent variables. The final model had acceptable fit to the data (*χ* ^2^ = 518.51, *df* = 265, *p* < .001; CFI = 0.95; TLI = 0.95; RMSEA = 0.05).

**Figure 2:**
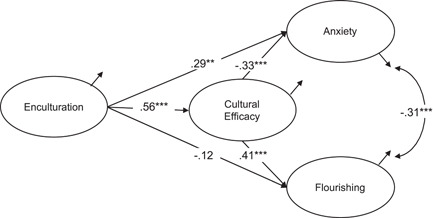
Structural equation model of enculturation, cultural efficacy, and mental health. *Note*: Controlling for sex, education, and location. Standardized coefficients. ***p < .001, **p < .01

The second step was to fit the structural model by including regression paths between our focal variables to assess our hypotheses (shown in Figure 2). To test the direct effects of our cultural variables on wellbeing, we regressed anxiety and flourishing on enculturation and cultural efficacy. To test the indirect effect of enculturation via cultural efficacy, we regressed cultural efficacy on enculturation. All of the latent variables were regressed on the control variables. We also allowed flourishing and anxiety to covary. The final measurement model had good model fit (*χ* ^2^ = 610.65, *df* = 348, *p* < .001; CFI = 0.94; TLI = 0.93; RMSEA = 0.04).

## RESULTS

Descriptive statistics and zero‐order correlations for the study variables are shown in Table 1. Respondents' mean flourishing score was 24 (of a possible 32). Respondents reported on average 5.31 anxiety symptoms, and 19% had 10 or more symptoms, which is the cut‐off for a positive anxiety screen (Spitzer et al., 2006). Turning to zero‐order correlations, flourishing and anxiety were significantly and negatively correlated. Cultural efficacy and enculturation were significantly and positively associated. As expected, both cultural efficacy and enculturation were positively correlated with flourishing, but enculturation was, paradoxically, positively associated with anxiety. Anxiety and cultural efficacy were negatively correlated, following our expectations. We examined these unadjusted associations in more depth using structural equation modeling, controlling for gender, location, and education.

Results of the structural equation modeling are shown in Table 2 and Figure 2. Being female was positively related to anxiety (*b* = 0.35, *p* < .001) but unrelated to the other focal variables. Living on reservation/reserve was positively associated with *cultural efficacy* (*b* = 0.25, *p* < .01) and negatively associated with flourishing (*b* = −0.21, *p* < .01). Having at least some postsecondary education was positively related to enculturation (*b* = 0.63, *p* < .01) and with flourishing (*b* = 0.19, *p* < .01). Enculturation was significantly, positively associated with *cultural efficacy* (*b* = 0.25, *p* < .001) and with anxiety (*b* = 0.15, *p* < .001), but was unrelated to flourishing (contrary to the significant zero‐order correlation observed in Table 1). *Cultural efficacy* was negatively associated with anxiety (*b* = −0.40, *p* < .001) and positively associated with flourishing (*b* = 0.41, *p* < .001). Explained variance for anxiety and flourishing was 13.5% and 19.7%, respectively.

**Table 2:** Structural equation model of enculturation, cultural efficacy, and mental health (*n* = 452)

	Latent response variables											
	Enculturation			Cultural efficacy			Anxiety			Flourishing		
	*b*	SE	*β*	*b*	SE	*β*	*b*	SE	*β*	*b*	SE	*β*
Sex	0.35*	.17	.23	−0.05	.07	−.07	0.34***	.09	.41	−0.04	.06	−.07
On versus off	0.17	.18	.11	0.25**	.09	.37	0.17	.09	.21	−0.21**	.07	−.35
Remote	0.83**	.29	.53	0.16	.13	.23	−0.19	.14	−.24	‐0.18	.10	−.29
Education	0.63**	.20	.40	0.05	.08	.07	0.01	.09	.01	0.19**	.06	.32
Enculturation				0.25***	.04	.56	0.15**	.04	.29	−0.05	.03	−.12
Cultural efficacy							‐0.40***	.10	−.33	0.41***	.10	.45
*R* ^2^	.07			.36			.14			.20		

*Note*: Sex coded 1 = female, 0 = male; on versus off coded 1 = on reservation/reserve, 0 = off reservation/reserve; remote location coded 1 = remote, 0 = rural; education coded 1 = at least some postsecondary education, 0 = no postsecondary education. Model fit statistics: *χ* ^2^ = 714.87, *df* = 348, *p* < .000; comparative fit index = 0.93; Tucker–Lewis index = 0.92; root mean square error of approximation = 0.05.

*** *p* < .001

** *p* < .01

* *p* < .05.

To test the effect of enculturation on mental health through its relationship with *cultural efficacy*, we used MODEL INDIRECT and decomposition of effects (Table 3). Enculturation had significant indirect effects on both measures of mental health via *cultural efficacy*. The indirect effect of enculturation on anxiety was −0.19 (*p* < .001), which reduced the direct effect of enculturation to nonsignificance (total effect = 0.11, *p* > .05) when accounting for *cultural efficacy*. There was also a significant indirect effect of 0.26 (*p* < .001) between enculturation and flourishing via *cultural efficacy*, which resulted in a positive and significant total effect (0.13, *p* < .05), despite a nonsignificant and negative direct effect.

**Table 3:** Standardized decomposition of effects of enculturation on mental health with bootstrapped standard errors and 95% confidence intervals (CIs)

	*β*	95% CI
Enculturation to anxiety		
Total effect	.11	−0.02, 0.20
Indirect effect via cultural efficacy	–.19***	−0.31, −0.08
Direct effect	.29***	0.14, 0.44
Enculturation to flourishing		
Total effect	.13*	0.01, 0.24
Indirect effect via cultural efficacy	.26***	0.12, 0.40
Direct effect	–.12	−0.30, 0.04

*Note*: Bootstrapped standard errors and 95% CI could not be estimated using maximum likelihood with robust standard errors; maximum likelihood used instead.

*** *p* < .001

** *p* < .01

* *p* < .05.

## DISCUSSION

In this study, measures of Indigenous enculturation were positively and directly associated with *cultural efficacy*. One interpretation of this finding is that the more an individual is culturally engaged (i.e., enculturated), the more confident they are being in cultural settings, centering culture in their roles and purpose, and seeking out and/or asking for cultural advice (i.e., cultural efficacy). This effect has the potential to proliferate enculturation. That is, the more we can provide consistent opportunities for cultural activity engagement, the more we can build confidence, a sense of belonging, and identity surrounding traditional ways/values for Indigenous peoples. The positive role of efficacy in healthy behaviors and activities has been supported by prior studies in terms of positive associations with physical activity (Olander et al., 2013), substance use treatment (Kadden & Litt, 2011), and among Indigenous populations, smoking cessation (Hendricks et al., 2014; Thomas et al., 2019). This body of literature, in tandem with our findings, signal the importance of broadening attention to the ongoing effects of historical trauma to include consideration of internal barriers to engaging culturally.

In stark contrast to community and Elder narratives that lean heavily on traditional Indigenous culture for sources of healing and strength (Kral et al., 2011), our structural equation models revealed that enculturation was associated with greater anxiety and was not directly associated with our positive mental health measure. In general, the state of the scientific literature with regard to Indigenous enculturation and health outcomes is mixed. For example, some studies report a negative or nonsignificant relationship between cultural variables and health outcomes (Walls et al., 2016), while others clearly demonstrate the protective effects of culture (Kading et al., 2015; Kaholokula et al., 2018; Mckinley et al., 2020). In light of these contrary findings, we posit that adequate measurement of the multiple dimensions and domains of Indigenous “culture” does not yet exist. There is a need for researchers to more consistently come together with Indigenous community members to unpack the operationalization of “culture” and the mechanisms at play that may clarify our understanding of culture as healing (Kagawa‐Singer et al., 2014).

In this vein, our novel measure of *cultural efficacy* was a critical variable for better understanding Indigenous wellbeing in the current analyses. First, *cultural efficacy* was associated with greater mental wellbeing, in alignment with previous research in Māori communities (Houkamau & Sibley, 2011). Further, and as hypothesized, the relationship between enculturation and mental wellbeing operated indirectly through *cultural efficacy*.

We believe that assessing *cultural efficacy* is one way to acknowledge the postcolonial context in which Indigenous culture and healing exist today. For example, the revitalization and reclamation of Indigenous languages and cultural practices is a process that evolves over time within communities and across one's own life course. Our results suggest that considering appraisal of one's own degree of cultural efficacy may be an important dimension of empirically documenting the complex pathways between Indigenous “culture” and health. This point is further underscored by the decomposition of effects of enculturation on mental health (Table 3). Notably, while the direct associations between enculturation and anxiety produced positive coefficients, the indirect effects via *cultural efficacy* were positive and total effects nonsignificant. Further, the positive and significant association between enculturation and flourishing existed only indirectly via *cultural efficacy*. Thus, concentrating community efforts to enhance participants' confidence and agency towards engaging in cultural activities beyond programming services may be a key mechanism through which the positive impact of culture‐based services and programming is realized. Our results also promote the critical role of Indigenous health care practitioners who may be able foster cultural efficacy by supporting their clients in their development of efficacy skills and welcoming their clients into cultural spaces; another reason to support Indigenous people entering healthcare careers.

### Limitations

As understanding of cultural mechanisms for health evolve, it is difficult to apply standard variables for assessing enculturation and Indigenous wellbeing. Community‐based participatory models of research are fairly new, and community input on these variables has been limited. Further, Indigenous voices and representations of their communities are only beginning to emerge amidst systemic oppression throughout the health, education, and research sectors. Our research team continues to develop and modify measurements of culture that make sense within an ever‐evolving context; while this may improve measurement validity, it is also challenging to fully utilize longitudinal data. In the current study, we used existing measures of anxiety and flourishing mental health as key outcomes. Our choice to include a state‐based measure of angst (i.e., the GAD‐7, which assesses anxiety symptoms in the past two weeks) seems appropriate given the dynamic, state‐based nature of the cultural efficacy framework as we have presented it. Further, CRCs reviewed each of these measures for comprehension and cultural validity before data collection.

### Future directions

We identify three specific recommendations for future research on culture and wellbeing with Indigenous Peoples. First, developmental processes of cultural efficacy vis‐à‐vis processes of enculturation remain an empirical question that needs further exploration. Second, expanded assessment of mental health outcomes is also of importance. For example, much has been written about cultural idioms of distress and distinctive understandings of mental health in Indigenous contexts, leaving room for measurement evaluation and expansion (Walls et al., 2019). Third, investing in community capacity to engage in research is important as studies investigate culture as a healing mechanism and as research teams build their own capacity to assess culture and health over time.

## CONCLUSION

Culture will always be at the foundation of Indigenous health, and colonization will always be considered a fundamental turning point interrupting Indigenous wellbeing. Evidence exists that supports the use of traditional foods, traditional medicines, spiritual activities, and community connectedness to improve wellbeing (Kading et al., 2015; Ullrich, 2019; Walls et al., 2016). However, because Indigenous peoples have some of the gravest health inequities in the world, substantial efforts to identify and support cultural healing opportunities is critical to alleviate the injustices of colonization and ongoing racial oppression. The current study examined a unique approach to understanding the healing powers of culture by considering the personal capacity of Indigenous individuals to engage culturally (i.e., enculturation) while acknowledging postcolonial personal barriers (i.e., lack of knowledge, cultural disconnection). Our findings suggest that increasing Indigenous people's degree of enculturation may in turn be associated with increases in *cultural efficacy*, which is linked to protective mental health effects. Specific programming may include establishing mentorship relationships with Elders and/or cultural knowledge keepers, increasing the transmission of Indigenous teachings through widespread culture‐based programs, and expanding ceremonial and cultural activity inclusion and support. Systemic investment in and policies to support Indigenous cultural initiatives are an important component for Indigenous community capacity for self‐healing.

## APPENDIX A DESCRIPTIVE STATISTICS AND FACTOR LOADINGS FOR LATENT VARIABLES

**Table:**

Item	Factor loading	Mean	SD	Range
*Enculturation*				
Traditional activities	0.51	3.26	3.18	0–17
Traditional language fluency	0.53	1.68	1.20	0–5
Spiritual activities checklist	0.77	4.27	3.26	0–13
Frequency of participation in spiritual activities	0.68	1.95	1.75	0–6
Importance of spiritual values	0.73	2.04	0.80	0–3
*Cultural efficacy*				
I feel confident about my ability to learn Anishinaabe cultural ways	0.52	2.92	0.85	0–4
Many of the things I do are to give back to Anishinaabe people	0.71	2.55	0.94	0–4
I am satisfied with my opportunities to engage in Anishinaabe cultural ways	0.47	2.61	0.95	0–4
I feel sure about who to ask to learn about Anishinaabe cultural ways	0.41	2.55	1.01	0–4
I want to be a role model for other Anishinaabe people	0.61	2.95	0.81	0–4
*Anxiety*				
Feeling nervous, anxious, or on edge	0.82	0.84	1.00	0–3
Not being able to stop or control worrying	0.81	0.76	0.96	0–3
Worrying too much about different things	0.82	0.89	0.96	0–3
Trouble relaxing	0.85	0.84	1.00	0–3
Being so restless that it is hard to sit still	0.78	0.66	0.95	0–3
Becoming easily annoyed or irritable	0.76	0.82	0.94	0–3
Feeling afraid as if something awful might happen	0.70	0.52	0.86	0–3
*Flourishing*				
I lead a purposeful and meaningful life	0.76	2.99	0.80	0–4
My social relationships are supportive and rewarding	0.76	2.92	0.76	0–4
I am engaged and interested in my daily activities	0.74	2.92	0.76	0–4
I actively contribute to the happiness and well‐being of others	0.67	3.05	0.67	0–4
I am competent and capable in the activities that are important to me	0.74	3.10	0.65	0–4
I am a good person and live a good life	0.77	3.11	0.72	0–4
I am optimistic about my future	0.62	3.03	0.77	0–4
People respect me	0.67	2.96	0.72	0–4
